# Mild cognitive dysfunction of caregivers and its association with care recipients’ end-of-life plans and preferences

**DOI:** 10.1371/journal.pone.0196147

**Published:** 2018-04-30

**Authors:** Keiko Kurita, Mark S. Lachs, Ronald D. Adelman, Eugenia L. Siegler, M. Cary Reid, Holly G. Prigerson

**Affiliations:** 1 Center for Research on End-of-Life Care, Weill Cornell Medicine, New York, New York, United States of America; 2 Division of Geriatrics and Palliative Medicine, Department of Medicine, Weill Cornell Medicine, New York, New York, United States of America; Taipei Veterans General Hospital, TAIWAN

## Abstract

Little is known about the association between cognitive dysfunction among informal caregivers and patients’ plans and preferences for patients’ end of life care. We report on the frequency of cognitive dysfunction among both patients and caregivers and examine associations between caregivers’ cognitive screening scores and end of life plans and preferences of patients with advanced cancer. The current sample was derived from a National Cancer Institute- and National Institute of Mental Health-funded study of patients with distant metastasis who had disease progression on at least first-line chemotherapy, and their informal caregivers (n = 550 pairs). The Pfeiffer Short Portable Mental Status, a validated cognitive screen, was administered to patients and caregivers. Patients were interviewed about their end of life plans and preferences. Logistic regression models regressed patients’ advance care planning and treatment preferences on caregivers’ cognitive screen scores. Patients’ cognitive screen scores were included as covariates. Most caregivers (55%) were spouses. Almost 30% of patients scored worse on the cognitive screen than their caregivers and 12% of caregivers scored worse than the patients. For each additional error that caregivers made on the cognitive screen, patients were more likely (AOR = 1.59, p = 0.002) to report that they preferred that everything possible be done to keep them alive and were less likely (AOR = 0.75, p = 0.04) to have a living will or a health care proxy/durable power of attorney. Worse caregiver cognitive screening scores were associated with higher likelihood of patients’ reporting that they wanted everything done to save their lives and a lower likelihood of having a living will or other type of advanced care plan. Future studies should confirm these findings in other populations and determine the mechanisms that may underlie the identified relationships.

## Introduction

Family and friends who serve as unpaid, informal caregivers of patients with serious illnesses assume various caregiving responsibilities ranging from assisting with activities of daily living (e.g., meal preparation, transportation) to accompanying them to medical appointments.[1] As patients approach death, caregiving responsibilities often expand to include representing patients’ wishes and making medical decisions on their behalf.[2,3]. These end-of-life (EoL) caregiving responsibilities are challenging because they often involve weighing complicated and suboptimal treatment alternatives, engender feelings of conflict, and carry long-term consequences. [4] Thus, especially at patients’ EoL, caregivers should be able to perform these cognitively demanding tasks. Because caregivers often influence the medical decisions of patients,[5,6] it is important to examine how cognitive impairment of caregivers may affect patient outcomes.

A few studies have shown that caregivers can have compromised cognitive function,[7–13] and that this impairment is associated with suboptimal patient care.[14] Studies examining the association between caregiver cognitive impairment with care at the EoL are lacking, however. In particular, mild cognitive dysfunction of caregivers may be more common but less obvious than severe impairment, as it allows for independent function without interference with daily life activities. Nonetheless, individuals who have mild dysfunction may have impairment in executive function, difficulties finding words, following directions, or staying on task. These impairments, in turn, may influence patients’ EoL preferences and plans in ways heretofore unrecognized. Thus we sought to examine the frequency of mild cognitive dysfunction in advanced cancer patients and their informal caregivers, and the influence of caregivers’ cognitive dysfunction on patient EoL preferences and care plans.

## Materials and methods

### Sample

The current sample was derived from Coping with Cancer, a National Cancer Institute- and National Institute of Mental Health-funded, longitudinal study of terminally ill patients with cancer and their informal caregivers to examine how psychosocial factors influence patients’ care and their caregivers’ bereavement adjustment. Patients were identified and recruited from 8 outpatient cancer clinics in the Northeast and Southwest United States.[15] The IRBs at Weill Cornell Medicine, Partners System (Dana-Farber Cancer Institute, Brigham and Women’s Hospital, Massachusetts General Hospital), Yale University School of Medicine, University of Texas Southwestern Medical Center, and Memorial Sloan-Kettering Cancer Center approved this study protocol.

To be eligible, patients were required to be > = 20 years of age with distant metastasis and have disease progression on at least first-line chemotherapy. In addition, they each had the participation of the person whom the patient nominated as providing most of their informal care. Additional requirements included adequate stamina to complete the study; fluency in English or Spanish; and a score of < = 6 on the Short Portable Mental Status (SPMS) exam.[16] In the current study, we include only patients and caregivers who made < = 4 errors on the SPMS to limit the sample to those having no worse than mild impairment and only information collected at baseline was used.

Patients and their primary informal caregiver were interviewed separately. A trained rater interviewed patients and caregivers and administered the SPMS.

### Measures

#### Cognitive screening

The SPMS, a validated, 10-item screen for cognitive impairment, was administered to both patients and caregivers.[16] According to the SPMS scoring guidelines, intact mental function was defined as the respondent having a score of 0 to 2, and mild impairment as scores of 3 to 4. The cognitive screening score for each participant was the number of errors made, adjusted for years of education. Thus, higher scores indicated more errors. Caregiver cognitive screening score was the main predictor variable.

#### Outcomes

Patients were asked, and medical chart review confirmed, if they had completed a Do Not Resuscitate (DNR) order; signed a living will; had a health care proxy (HCP) and/or durable power of attorney (DPA) for health care; and acknowledged their health status as terminal. Patients were also asked about preferences including whether they preferred “extending life as much as possible, even if it meant more pain and discomfort” or whether they wanted “everything possible to keep you alive even if you were going to die in a few days anyway.” Finally, patients were asked if they would accept chemotherapy, a “breathing machine,” or a feeding tube to be kept alive. Outcomes were dichotomous (Yes/No).

#### Statistical analysis

In the base models, the odds of the caregivers’ cognitive scores on EoL outcomes were estimated using logistic regression. The adjusted models also included the patients’ cognitive scores to control for effects of patient cognitive scores on EoL care, as previously reported. [17] SAS 9.4 was used, hypothesis tests were two-sided, and p < 0.05 was considered statistically significant.

## Results

Table 1 characterizes the sample (n = 550 pairs). 279 (55.1%) were spouses or partners of patients, 112 (22.1%) were their sons or daughters, 38 (7.5%) were their sibling, 35 (6.9%) were another relative, and the remaining were friend, parent, or other. For 29.6% of the pairs, patients made more errors on the SPMS than their caregivers and for 12.3% of the pairs, caregivers made more errors than their patients (Table 2). Patients and caregivers scores were not significantly associated with each other (r = 0.03; p = 0.54).

**Table 1:** Sample characteristics (n = 550 pairs).

	Patients (n = 550)	Caregivers (n = 550)
Mean age (SD), *years*	59.4 (13.1)	53.0 (14.2)
Education,^a^ *years*	12.7 (3.9)	13.5 (3.4)
Female,^a^ *n (%)*	278 (50.7%)	393 (71.5%)
Race/ethnicity, ^a^ *n (%)*		
White	383 (69.9%)	375 (68.6%)
Other	165 (30.1%)	172 (31.4%)
Cognitive screening, mean errors (SD)	0.6 (0.9)	0.2 (0.6)
Cognitive screening, number of errors *n (%)*		
0 errors	365 (66.4%)	462 (84.0%)
1 error	87 (15.8%)	46 (8.4%)
2 errors	76 (13.8%)	36 (6.6%)
3 errors	16 (2.9%)	6 (1.1%)
4 errors	6 (1.1%)	

Participants were screened using the SPMS. Errors were adjusted based on participant’s level of education, as per scoring guidelines

^a^n = 549 for patients

**Table 2:** Relative number of errors made in patient-caregiver dyads (n = 550 pairs).

	n	frequency %
Patients made 4 more errors than caregivers	5	0.9%
Patients made 3 more errors than caregivers	11	2.0%
Patients made 2 more errors than caregivers	72	13.1%
Patients made 1 more error than caregivers	75	13.6%
Patients and caregivers made the same number of errors	320	58.2%
Caregivers made 1 more error than patients	40	7.3%
Caregivers made 2 more errors than patients	24	4.4%
Caregivers made 3 more errors than patients	3	0.6%

The patient and caregiver cognitive scores were differentially associated with EoL plans and preferences (Table 3). For each additional error caregivers made on the SPMS, patients were more likely (AOR = 1.59) to report that they prefer doing “everything to live even if dying in a few days anyway” and were less likely (AOR = 0.75) to have completed a living will or a HCP/DPA.

**Table 3:** Associations between caregiver scores on a cognitive screen and patient care (n = 550).

	Base Model		Full Model			
	Caregiver Errors		Patient Errors		Caregiver Errors	
	OR (95% CI)	P-value	OR (95% CI)	P-value	OR (95% CI)	P -value
**Patients’ plans**						
Has DNR^a^	1.06 (0.81, 1.40)	0.66	1.23 (1.02, 1.48)	0.03	1.06 (0.80, 1.40)	0.70
Has living will^a^	0.75 (0.57, 0.99)	0.04	1.00 (0.83, 1.21)	0.83	0.75 (0.57, 0.99)	0.04
Has HCP, DPA^a^	0.75 (0.57, 0.99)	0.04	1.02 (0.85, 1.23)	0.70	0.75 (0.57, 0.99)	0.04
Acknowledges terminal illness^b^	0.85 (0.64, 1.15)	0.30	1.21 (1.00, 1.48)	0.05	0.84 (0.63, 1.13)	0.26
**Patients’ preferences**						
Extend life over pain relief^c^	1.00 (0.75, 1.38)	0.93	0.85 (0.67, 1.07)	0.16	1.02 (0.75, 1.34)	0.90
Do everything to live even if dying in a few days^d^	1.60 (1.19, 2.16)	0.002	1.15 (0.92, 1.43)	0.21	1.59 (1.18, 2.15)	0.002
Would accept chemotherapy to be kept alive^d^	0.85 (0.62, 1.17)	0.32	0.77 (0.63, 0.95)	0.01	0.86 (0.63, 1.18)	0.34
Would accept breathing machine to be kept alive^d^	1.18 (0.88, 1.60)	0.28	0.91 (0.73, 1.13)	0.39	1.19 (0.88, 1.60)	0.27
Would accept feeding tube to be kept alive^e^	1.29 (0.98, 1.71)	0.07	0.80 (0.65, 0.99)	0.04	1.30 (0.98, 1.73)	0.07

DNR = do not resuscitate; HCP = Health care proxy; DPA = Durable Power of Attorney.

Models were based on missing responses resulting in the following sample sizes:

^a^n = 534;

^b^n = 518;

^c^n = 491;

^d^n = 531;

^e^n = 533.

## Discussion

Nearly one-third of patients with advanced cancer had worse cognitive screening scores compared to their caregivers, but at least one in nine caregivers—who were identified by patients as the person providing most of their informal care—had worse scores than patients. Worse caregiver cognitive screening scores were associated with patients reporting that they would “do everything to live even if dying in a few days.” Additionally, worse caregiver cognitive screening scores were associated with fewer completions of living will and healthcare power of attorney orders. Patient and caregiver cognitive screening scores were not significantly associated with one another and differentially were associated with patients’ EoL plans.

Cognitive dysfunction has been extensively documented among patients with advanced cancer [18] so it was expected that a proportion of patients would have worse cognitive screening scores than their caregivers. Surprisingly, we also found that in 12% of the patient-caregiver dyads, caregivers scored worse than their patients. Although cognitive deficits in caregivers have been less frequently examined, its presence has been reported in those caring for patients with serious, long-term illnesses such as dementia or stroke. [11,12] The physiological and psychosocial mechanisms that explain cognitive dysfunction in caregivers such as cortisol levels, stress, and health habits,[19] may also exist in the caregivers of patients with advanced cancer in this study. While these results suggest that even middle-age caregivers could experience mild cognitive dysfunction, most were considered mentally intact and the effects may be temporary. It is possible that caregivers would make one or two careless mistakes on a cognitive screen during this highly stressful, intense period when providing for a family member with advanced cancer. The range of cognitive dysfunction in caregivers and patients without dementia leaves open the possibility that some patients may have suspected or been aware of mild cognitive dysfunction in their primary caregiver, which may have had some influence in their plans and preferences. These findings show that even among patients and caregivers who were at most mildly impaired, cognitive screening scores of patients and caregivers were differentially associated with patient plans and preferences. Furthermore, in older patient-caregiver dyads, higher proportions of mild cognitive dysfunction and other associations may be uncovered.

The second main finding builds on our previous study that demonstrated that cognitive dysfunction in patients is associated with caregivers’ preferences.[20] In the current study we found a significant association between worse caregiver cognitive scores and patients’ reported preference to do everything to save their lives even if they were dying in a few days. This may be the result of a strong co-dependency—such as if the patient assisted in caring for the primary caregiver who was mildly impaired or if a caregiver was so distraught and anxious anticipating the patient’s death.[21–23] This finding was also consistent with the likelihood that patients who had caregivers with worse cognitive scores had completed a living will, HCP, or DPA. Despite their reported preferences, patients did not direct how this would change their care, as there was no significant association with actual life-sustaining treatments received or procedures or completion of a DNR order.

Limitations of this investigation include that it is a secondary analysis of data from a study that excluded patients with significant cognitive impairment. In addition, data collection was completed in 2008 so findings may not reflect plans and preferences of current patients with advanced cancer. Strengths of this study include the assessment of the physical and mental health and EoL care preferences in advanced cancer patients and their caregivers to examine issues relevant to patient EoL care.

Given the regularity with which many provider groups (e.g., geriatricians, hospitalists, intensivists, oncologists) care for seriously ill patients with caregivers who have cognitive dysfunction at levels worse than the patients themselves, these findings appear to shed light on difficult, common clinical situation. If a caregiver’s ability to attend to information, think through problems, and remember instructions is compromised, our results suggest that this has ramifications for the patients’ EoL care plans. Our findings suggest a need for clinicians to be more aware of potential mild cognitive dysfunction in patients’ primary informal caregivers and proactively provide education, decisional aids, and other resources to ensure that patient’s EoL care is consistent with informed preferences of both the caregivers and the patients for whom they care. Successful EoL care planning does not have to result in completed DNR orders or living wills. However, it should allow decision-making and goals of care discussions with the appropriate family members in a comfortable, safe setting using language and terms that are understandable for all involved. Future research, including with samples that include a wider range of cognitive dysfunction, is needed to improve understanding and care in this challenging, yet common, EoL care context.
